# Bariatric Procedures in Older Adults in the United States: Analysis of a Multicenter Database

**DOI:** 10.3390/geriatrics4020032

**Published:** 2019-04-21

**Authors:** Priya Mendiratta, Neeraj Dayama, Gohar Azhar, Pallavi Prodhan, Jeanne Y. Wei

**Affiliations:** 1 Department of Geriatrics, Reynolds Institute on Aging, College of Medicine, Department of Geriatrics University of Arkansas Medical Sciences 4301 W. Markham Street, Little Rock, AR 72205, USA; AzharGohar@uams.edu (G.A.); pprodhan7@gmail.com (P.P.); WeiJeanne@uams.edu (J.Y.W.); 2 Department of Health Policy and Management, College of Public Health-University of Arkansas Medical Sciences, Little Rock, AR 72205, USA; NNdayama@uams.edu

**Keywords:** older adults, bariatric procedures, outcomes

## Abstract

Background*:* Bariatric procedures help reduce obesity-related comorbidities and thus improve survival. Clinical characteristics and outcomes after bariatric procedures in older adults were investigated. Methods*:* A multi-institutional Nationwide Inpatient Sample (NIS) database was queried from years 2005 through 2012. Older adults >60 years of age with procedure codes for bariatric procedures and a diagnosis of obesity/morbid obesity were selected to compare clinical characteristics/outcomes between those undergoing closed versus open procedures and identify risk factors associated with in-hospital mortality and increased hospital length of stay (LOS). Results*:* Over the study period, 79,122 bariatric procedures were performed. Those undergoing open procedures compared to closed procedures had a higher in-hospital mortality (0.8% vs. 0.2%) and a longer hospital LOS (4.8 days vs. 2.2 days). Risk factors significantly associated with in-hospital mortality were open procedures, the Western region, and the Elixhauser comorbidity index. Risk factors associated with increased LOS were Medicaid insurance type, an open procedure, a higher Elixhauser comorbidity score, a required skilled nursing facility (SNF) discharge, and died in hospital. Conclusion*:* Closed bariatric procedures are increasingly being preferred in older adults, with a four-fold lower mortality compared to open procedures. Besides choice of procedure, the presence of specific comorbidities is associated with increased mortality in older adults.

## 1. Introduction

Over the past 25 years, there has been an exponential rise in the prevalence of obesity in the United States. Currently, about 41% of older adults above 60 years of age are considered obese [1]. Bariatric procedures are recommended for adults with morbid obesity (Class 3 obesity) or in those with a body mass index (BMI) ≥ 35 kg/m^2^ (Class 2 obesity) in the presence of high-risk comorbid conditions [2]. With longer life expectancy, coupled with the increased prevalence of obesity and a concomitant decrease in procedure-related risks of complications, greater numbers of older patients are seeking bariatric procedures [3,4]. For these patients, bariatric procedures help reduce many obesity-related comorbidities and thus improve overall survival.

Although a large body of literature informs bariatric procedure utilization in younger adults, the clinical characteristics, temporal case volume trends, and outcomes of bariatric procedures in older adults (>60 years) is still limited [5,6,7,8,9,10] to single-center reports with smaller sample sizes. Furthermore, older age is itself associated with age-related comorbidities that could potentially augment the risk profile of bariatric procedures in older adults. Therefore, in this study, we investigated clinical characteristics, temporal trends in case volume, and outcomes after bariatric surgery in older adults (≥60 years of age).

## 2. Methods

### 2.1. Data and Sample

Retrospective data were pooled from the 2005 to 2012 Nationwide Inpatient Sample (NIS) database, which is sponsored by the Agency for Healthcare Research and Quality as part of the Healthcare Cost and Utilization Project (HCUP). The NIS database is the largest publicly available all-payer inpatient care database in the United States, representing a 20% stratified sample of all U.S. community hospitals. The HCUP was used to create national estimates for trends analyses [11,12].

All patients aged 60 years or older with an “International Classification of Disease, 9th Revision, Clinical Modification” procedure code of laparoscopic gastric bypass (44.38), open gastric bypass (44.31 and 44.39), laparoscopic gastric band (44.95), and laparoscopic gastroplasty (44.68) with principal diagnosis codes of obesity and morbid obesity (278.0, 278.01, 278.8, and 278.1) were selected. Patients transferred to another short-term hospital were excluded to avoid double counting. Approval for use of the NIS patient-level data in this study was obtained from the Institutional Review Board of the University of Arkansas for Medical Sciences.

### 2.2. Study Variables

The data variables investigated were categorized as patient-specific factors (age, gender, race, payer type), obesity-specific factors (comorbidities), and procedure-specific factors (operative technique (laparoscopic versus open type) and outcome variables). The outcome measures investigated were in-hospital mortality and hospital length of stay after undergoing a bariatric procedure. The study hypotheses were that (a) older age, (b) specific racial groups, and (c) the presence of chronic medical conditions were not associated with increased mortality after a bariatric procedure.

### 2.3. Data Analyses

Patient and hospital characteristics are presented as relative frequencies and percentages for each independent variable. All values for continuous variables are expressed as means ± standard deviations. *T*-statistics were calculated to analyze the overall trend of each independent variable during the study period. Univariate and multivariate regression analyses were performed to identify independent predictors of in-hospital mortality and hospital length of stay. A generalized linear model with a logit link and gamma distribution was fitted for right-skewed cost data as a function of all independent variables. [7] Statistical evaluations were performed using SAS version 9.2 (SAS Institute, Cary, NC, USA), and statistical significance was defined as *p* < 0.05.

## 3. Results

**Figure 1:**
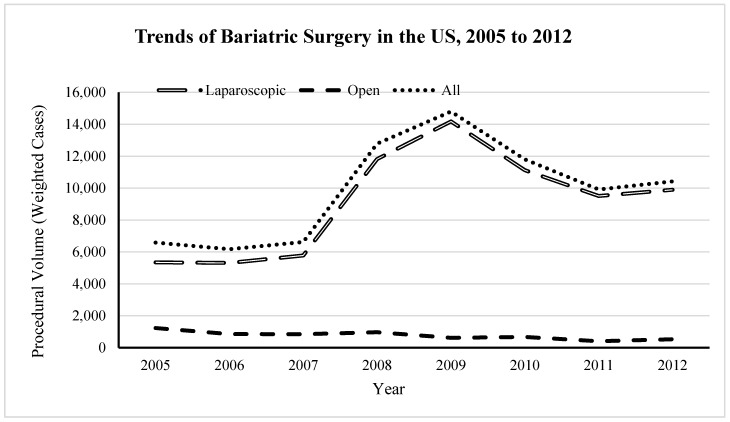
Temporal trends of bariatric procedures in the United States in older adults, 2005–2012.

Over the study period, 79,122 bariatric procedures were performed. The majority of the procedures were closed procedures (92.2%). Figure 1 shows the statistically significant temporal trends of bariatric procedures over the study period. The numbers of open and closed procedures peaked in 2008 and 2009, respectively.

Table 1 shows the clinical characteristics of the study population and compares subjects undergoing closed versus open procedures. Subjects undergoing open procedures compared to those undergoing closed procedures were more likely to have the following characteristics: Older age (63.3 years vs. 42.2 years), female gender (73.7% vs. 70.6%), care from rural hospitals (7% vs. 3.8%), Medicaid (2.8% vs. 1.5%), private insurance (48.6% vs. 42.7%), a higher mean Elixhauser comorbidity index (2.5 vs. 2.4), chronic liver disease (13.8% vs. 9.8%), a longer hospital length of stay (LOS) (4.8 days vs. 2.2 days), a required SNF discharge (4.9% vs. 0.8%), and higher in-hospital mortality (0.8% vs. 0.2%). In contrast, those undergoing closed procedures compared to those undergoing open procedures were more likely to have the following characteristics: Care from an urban nonteaching hospital (44.3% vs. 35.4%), care in the Western region (25.7% vs. 15.7%), Medicare (48.2% vs. 40%), hyperlipidemia (57.2% vs. 50.2%), hypertension (77.7% vs. 73.9%), and a routine discharge (95.4% vs. 85%).

### 3.1. Risk Factors Analysis

Nonsurvivors, when compared to survivors (Table 2), were more likely to have the following characteristics: Having undergone an open procedure (26.3% vs. 7.7%), an older mean age (65.1 years vs. 64.1 years), a higher mean Elixhauser index (3.2 vs. 2.9), congestive heart failure (9.4% vs. 3.6%), COPD (33.1% vs. 21%), peripheral artery disease (10.2% vs. 1.5%), and a higher mean hospital LOS (25.9 d vs. 2.3 d). Risk factors significantly associated with in-hospital mortality (multivariable analysis, Table 3) for subjects undergoing a bariatric procedure were open procedures (odds ratio (OR) 4.1; 95% confidence interval (CI) 1.8–9.1), the Western region (OR 3.3, 95% CI 1.04–10.3), and an Elixhauser comorbidity index (OR 1.5, 95% CI 1.1–2.02).

### 3.2. Hospital Length of Stay

The risk factors associated with an increased length of stay were Medicaid insurance type (OR 1.13, 95% CI 1.00–1.27), an open procedure (OR 1.74, 95% CI 1.62–1.88), a higher Elixhauser comorbidity score (OR 1.10, 95% CI 1.08–1.12), a required SNF discharge (OR 2.73, 95% CI 2.17–3.44), and nonsurvivors (OR 4.87, 95% CI 2.35–10.12) (Supplemental Materials, Table S1).

## 4. Discussion

Our study utilized a large multicenter national database to provide an in-depth analysis of trends and outcomes of bariatric procedures among obese older adults. In this population, our results highlighted (a) the temporal trend in bariatric procedures, (b) a comparison of the clinical characteristics and outcomes between those undergoing closed versus open procedures, and (c) an identification of the unique risk factors associated with in-hospital mortality and increased hospital LOS.

The procedure-related temporal trends indicated that open procedure rates in older adults declined from 18.6% in 2005 to 5.1% in 2012. This trend was similar to that noted in all adult populations, where the frequency of open procedures declined from 14% [13] in the 2006–2008 era to 2.9% in 2012 [14]. This indicates an increasing preference for closed procedures, given their efficacy and better outcomes over open procedures. Previous studies have shown that older age is a risk factor for mortality [15,16,17] after bariatric procedures. In a study of 1067 consecutive patients, Livingston et al. [15] reported that patients 55 years and older had a three-fold greater mortality from gastric bypass surgery compared to younger patients. However, within the older adult population, we found that older age (>71 years), when compared to 60–70 years of age, did not confer any additional mortality risk. Surprisingly, in our study, age > 71 years was also associated with a shorter hospital LOS compared to those between the ages of 60 and 70 years. Additionally, despite a higher prevalence of obesity in blacks and Hispanics compared to whites [1], we did not find any differences in mortality among these racial groups after bariatric procedures.

We found that the type of bariatric procedure performed (open versus closed) in older adults was an independent risk factor for in-hospital mortality. Closed procedures were associated with a four-fold lower mortality rate (0.2%) compared to open procedures (0.8%). These rates were similar to those that have been reported previously [18,19]. Nguyen et al. [13] indicated a mortality of 0.06% and 0.03% for laparoscopic gastric bypass and gastric banding, respectively, in a cohort of adults of all ages undergoing bariatric procedures between 2006 and 2008. Similarly, to our report, mortality was significantly higher for open procedures (0.52%). In a more recent report in adults, the majority of which were patients undergoing (97%) closed procedures, the in-hospital mortality ranged from 0.10% in 2010 to 0.07% in 2012 [14]. These temporal trends in mortality outcomes in recent decades have been an improvement over earlier outcomes: Mortality from closed procedures in 1998 was 0.8%, but it decreased to 0.5% by 2002 [20]. Similarly, Lancaster et al. [21] analyzed the American College of Surgeons National Surgical Quality Improvement Program (ACS-NSQIP) and similarly reported a significantly greater 30-day mortality after open versus laparoscopic gastric bypass (0.79% vs. 0.17%, respectively). However, in contrast to previous studies, where adult populations of all ages were studied, our investigation uniquely focused on older adults only. The overall improvement in mortality outcomes in older adults are multifactorial and possibly reflect increasing experience with greater case volumes, a broader adoption of the laparoscopic approach, appropriate fellowship training programs, and overall improvement in perioperative care. Surprisingly, in our study, we noted that patients undergoing open procedures had a higher Elixhauser comorbidity index in comparison to those undergoing closed procedures. It is unknown why open procedures were performed in those with more comorbidities. It can only be speculated that physician preference for open procedures over closed procedures may have been dictated in part by their limited training in laparoscopic procedures in earlier eras, and thus there was a propensity for a surgeon to perform open procedures even on those with increased comorbidities. These differences between open and closed procedures were undoubtedly related to the overall invasiveness of the open procedures. Nevertheless, this finding highlights the importance of informed procedural choice for older adults who have decreased physiological reserves and more associated comorbidities. Our report highlights the need to individualize procedural choice on a case-by-case basis, given a higher risk of mortality with open procedures in older adults.

Comorbidity, as indicated by the Elixhauser comorbidity score, was associated with increasing mortality and hospital LOS. However, none of the specific comorbid conditions investigated conferred a higher risk of mortality. These results were similar to those reported by Nguyen et al. [13]. However, in contrast, Carbonell et al. [22] did not find a correlation between comorbidities and greater mortality. In addition, contrary to our findings, Fernandez et al. [23] found hypertension to be predictive of greater perioperative mortality in a multivariate analysis of 2011 patients who underwent open and laparoscopic gastric bypass. When offering bariatric procedures to older adults, it is thus important to risk-stratify patients based on their comorbidities, and the use of well-validated preoperative risk profile scoring systems may further help about the impact of comorbidities among older adults as well [24].

There were limitations to this study. The NIS database is compiled from discharge abstract data and is limited to in-hospital stays without outpatient follow-up data. For example, deaths that occurred after discharge would not have been captured in this database, an underestimate of overall mortality. Many factors, such as other disparities not included in the analysis, the surgical volume of operations, the provider level, and clustering of cases/outcomes within hospitals, which may have affected mortality and are sources of bias in such a study, were not analyzed in this study. Last, we recognize the limitation of the dataset in gleaning reasons on what informed the choice of bariatric procedure in older adults. Information regarding the decisions relating to choose by surgeons and patients/their families was not available in the NIS database. Our dataset included a study period until 2012. It would have been important to include recent data. However, funding issues limited our ability to procure recent data. Despite these limitations, this novel study is the largest to date in evaluating factors predictive of mortality in bariatric surgery, specifically in older adult patients.

## 5. Conclusions

Bariatric surgery is increasingly being offered to older adults with a preference for closed procedures. Open procedures are associated with a four-fold increased mortality compared to closed procedures. Besides choice of procedure (open vs. closed), the presence of specific comorbidities is associated with increased mortality in older adult patients. Our report stresses the need to individualize bariatric surgery choice based on a case-by-case evaluation of the type of procedure offered and the presence of comorbidities.

## Figures and Tables

**Table 1:** Comparison of clinical characteristics between patients undergoing open versus closed bariatric procedures. LOS: Length of stay.

Characteristic	Total	Laparoscopic	Open	*p*-Value
	*n* = 79,122	*n* = 72,696	*n* = 6153	
Demographics				
Age	64.16 (7.7)	64.23 (7.8)	63.29 (6.6)	<0.0001
Age 60 to 70	74,556	68,560 (92)	5997 (8)	0.0002
Age 71 to 80	4565	4409 (96.6)	157 (3.4)	0.0002
Female gender	56,025	51,493 (92)	4532 (8)	0.0315
Race				
White	57,970	53,673 (92.6)	4297 (7.4)	0.4639
Black	4592	4212 (91.7)	379 (8.3)	0.3426
Hispanic	3110	2942 (94.6)	168 (5.4)	0.1568
Insurance type				
Private	34,150	31,169 (91.3)	2981 (8.7)	0.0129
Medicare	37,611	35,152 (93.5)	2460 (6.5)	0.0004
Medicaid	1239	1069 (86.3)	170 (13.7)	0.0022
Hospital type				
Rural	3207	2777 (86.6)	429 (13.4)	0.0173
Urban Nonteaching	34,468	32,287 (93.7)	2180 (6.3)	0.0474
Urban teaching	40,929	37,413 (91.4)	3516 (8.6)	0.181
Hospital location				
Northeast	16,306	14,667 (89.9)	1639 (10.1)	0.0772
Midwest	17,688	16,170 (91.4)	1518 (8.6)	0.4552
South	25,386	23,355 (92)	2031 (8)	0.8165
West	19,742	18,777 (95.1)	965 (4.9)	0.001
Comorbidities				
Elixhauser comorbidity index	2.39 (2.8)	2.38 (2.8)	2.5 (3.1)	<0.0001
CHF (Congestive heart failure)	2831	2583 (91.2)	248 (8.8)	0.466
Hypertension	61,225	56,677 (92.6)	4547 (7.4)	0.0025
COPD (Chronic obstructive pulmonary disease)	16,596	15,270 (92)	1326 (8)	0.6533
Diabetes	40,327	37,248 (92.4)	3079 (7.6)	0.49
Peripheral vascular disease	1223	1112 (91)	111 (9)	0.4474
Renal failure	3141	2874 (91.5)	268 (8.5)	0.4679
Chronic liver disease	7972	7121 (89.3)	851 (10.7)	0.0221
Apnea	13,097	11,971 (91.4)	1126 (8.6)	0.1771
Hyperlipidemia	44,839	41,749 (93.1)	3091 (6.9)	<0.0001
Smoking	1333	1230 (92.3)	103 (7.7)	0.9856
Outcomes				
Died	189	140 (73.7)	50 (26.3)	<0.0001
LOS mean (Length of stay)	2.39 (8.52)	2.16 (7.1)	4.80 (18.3)	<0.0001
Routine discharge	74,801	69,573 (93)	5229 (69.9)	<0.0001
SNF (Skilled nursing facility) discharge	878	577 (65.7)	301 (34.3)	<0.0001

**Table 2:** Comparison of survivors and nonsurvivors among patients undergoing bariatric procedures.

Variables	Total	Alive	Dead	*p*-Value
	79,122	*n* = 78,932	*n* = 189	
Age	64.16 (7.1)	64.15 (7.7)	65.14 (6.9)	<0.0001
Age 60 to 70	74,556	74,380 (99.8)	176 (0.2)	0.7371
Age > 71	4565	4552 (99.8)	13 (0.2)	0.7371
Female gender	56,025	55,891 (99.8)	134 (0.2)	0.997
Race				
White	57,970	57,845 (99.8)	125 (0.2)	0.3076
Black	4592	4573 (99.5)	18 (0.5)	0.2703
Hispanic	3110	3094 (99.5)	15 (0.5)	0.1783
Insurance type				
Private	34,150	34,089 (99.8)	61 (0.2)	0.1492
Medicare	37,611	37,498 (99.7)	114 (0.3)	0.1
Medicaid	1239	1234 (99.6)	5 (0.5)	0.6671
Hospital type				
Rural	3207	3203 (99.9)	4 (0.1)	0.4298
Urban nonteaching	34,468	34,394 (99.8)	74 (0.2)	0.5688
Urban teaching	40,929	40,818 (99.7)	111 (0.3)	0.3593
Hospital location				
North East	16,306	16,284 (99.9)	22 (0.1)	0.1427
M West	17,688	17,644 (99.8)	44 (0.2)	0.9143
South	25,386	25,310 (99.7)	76 (0.3)	0.288
West	19,742	19,694 (99.8)	48 (0.2)	0.959
Type of procedure				
Laparoscopic/closed	72,969	72,829 (99.8)	140 (0.2)	<0.0001
Open procedure	6153	6103 (99.1)	50 (0.9)	<0.0001
Comorbidity				
Elixhauser comorbidity index	2.39 (2.8)	2.9 (2.8)	3.2 (3.8)	<0.0001
CHF	2831	2813 (99.3)	18 (0.7)	0.0381
Hypertension	61,225	61,146 (99.9)	79 (0.1)	<0.0001
COPD	16,596	16,533 (99.6)	63 (0.4)	0.0423
Apnea	13,097	13,083 (99.8)	14 (0.2)	0.1282
Smoking	1333	1325 (99.4)	8 (0.6)	0.1954
Renal failure	3141	3114 (99.1)	27 (0.9)	0.0006
Chronic liver disease	7972	7949 (99.7)	23 (0.3)	0.7113
Diabetes	40,327	40,247 (99.8)	80 (0.2)	0.3117
Peripheral vascular disease	1223	1204 (98.5)	19 (1.5)	<0.0001
Hyperlipidemia	44,839	44,784 (99.9)	55 (0.1)	0.0007
Length of stay	2.36 (8.7)	2.31 (6.3)	25.88 (109.2)	<0.0001

**Table 3:** Risk factor analysis for in-hospital mortality among patients undergoing bariatric procedures.

Variables	Univariate				Multivariate			
	OR	95% CI		*p*-Value	OR	95% CI		*p*-Value
Age								
Age 60 to 70	0.82	0.26	2.6	0.74	Reference			
Age > 71	1.22	0.38	3.85	0.74	1.00	0.26	3.93	1.00
Female gender	1.00	0.51	1.94	1.00	0.77	0.36	1.64	0.50
Race								
White	0.65	0.28	1.5	0.31	Reference			
Black	1.8	0.62	5.17	0.28	2.05	0.68	6.21	0.21
Hispanic	2.21	0.67	7.23	0.19	2.63	0.81	8.58	0.11
Insurance type								
Private	0.62	0.33	1.19	0.15	Reference			
Medicare	1.67	0.9	3.12	0.11	1.34	0.65	2.76	0.43
Medicaid	1.54	0.21	11.32	0.67	1.39	0.17	11.6	0.76
Hospital type								
Rural	0.47	0.07	3.19	0.44	Reference			
Urban nonteaching	0.83	0.44	1.56	0.57	1.11	0.14	8.99	0.92
Urban teaching	1.34	0.72	2.5	0.36	1.37	0.16	11.74	0.78
Hospital location								
North East	0.5	0.2	1.29	0.15	Reference			
Mid West	1.04	0.52	2.09	0.91	2.68	0.75	9.66	0.13
South	1.42	0.74	2.72	0.29	2.45	0.77	7.75	0.13
West	1.02	0.48	2.16	0.96	3.23	1.02	10.15	0.05
Type of procedure								
Laparoscopic/closed					Reference			
Open procedure	4.25	2.11	8.56	0.00	4.03	1.81	8.95	0.00
Comorbidity								
Elixhauser comorbidity index	1.52	1.19	1.94	0.00	1.76	1.29	2.42	0.00
CHF	2.81	1.01	7.84	0.05	0.82	0.19	3.61	0.79
Hypertension	0.21	0.11	0.39	0.00	0.17	0.08	0.36	0.00
COPD	1.87	1.01	3.47	0.05	0.77	0.35	1.68	0.51
Apnea	0.41	0.13	1.34	0.14	0.34	0.08	1.55	0.16
Smoking	2.52	0.59	10.7	0.21	2.91	0.63	13.56	0.17
Renal failure	4.12	1.7	9.97	0.00	0.37	0.1	1.37	0.14
Chronic liver disease	1.23	0.4	3.78	0.71	0.73	0.22	2.48	0.62
Diabetes	0.71	0.37	1.38	0.31	0.66	0.31	1.39	0.27
Peripheral vascular disease	7.37	2.67	20.36	0.00	2.21	0.37	13.33	0.39
Hyperlipidemia	0.31	0.16	0.63	0.00	0.27	0.12	0.59	0.00

## Supplementary Materials

The following are available online at https://www.mdpi.com/2308-3417/4/2/32/s1, Table S1: Risk factor analysis (univariate and multivariate) for hospital length of stay among patients undergoing a bariatric procedure.
