# Supplementary material for: Bariatric Procedures in Older Adults in the United States: Analysis of a Multicenter Database
Source: Geriatrics (Basel). 2019 Apr 21;4(2):32. doi: 10.3390/geriatrics4020032 (PMC6631888; doi:10.3390/geriatrics4020032)
Supplement: Supplementary file 1 [file geriatrics-04-00032-s001.pdf]

Supplemental Table 1: Risk factor analysis (univariate and multivariate) for hospital length of stay among patients undergoing a bariatric procedure

**Variables**	**Univariate Analysis**				**Multivariate analysis**			
	IRR	95% CI		p-value	IRR	95% CI		p-value
Age								
Age 60 to 70	1.21	1.10	1.34	0.00	Reference			
Age > 71	0.83	0.75	0.91	0.00	0.89	0.82	0.96	0.00
Female gender	1.08	1.02	1.14	0.01	1.03	0.99	1.06	0.11
Race								
White	0.95	0.88	1.03	0.22	Reference			
Black	1.14	1.01	1.28	0.03	1.06	0.99	1.13	0.10
Hispanic	1.03	0.91	1.15	0.67	1.00	0.93	1.08	0.97
Insurance type								
Private	1.02	0.96	1.08	0.50	Reference			
Medicare	1.00	0.95	1.06	0.97	0.95	0.91	0.98	0.01
Medicaid	1.28	1.09	1.50	0.00	1.13	1.00	1.27	0.04
Hospital type								
Rural	1.19	0.93	1.53	0.17	Reference			
Urban-Non Teaching	0.89	0.81	0.98	0.02	0.94	0.77	1.15	0.55
Urban Teaching	1.09	1.00	1.20	0.06	0.99	0.82	1.20	0.92
Hospital location								
N East	1.02	0.92	1.14	0.71	Reference			
M West	1.10	1.00	1.22	0.05	1.11	0.99	1.25	0.07
South	0.98	0.89	1.08	0.71	1.02	0.94	1.11	0.57
West	0.91	0.82	1.02	0.09	1.09	0.99	1.20	0.07
Procedure type								
Closed					Reference			
Open surgery	2.22	1.99	2.48	0.00	1.74	1.62	1.88	0.00
Co-morbidities								
Elixhauser comorbidity score	1.10	1.07	1.13	0.00	1.11	1.09	1.13	0.00
CHF	1.50	1.29	1.73	<0.001	1.06	0.97	1.16	0.21
Hypertension	0.72	0.67	0.78	<0.001	0.82	0.78	0.86	0.00
COPD	1.07	1.03	1.13	0.00	0.92	0.88	0.97	0.00
Apnea	0.92	0.88	0.96	<0.001	1.00	0.96	1.03	0.84
Smoking	0.95	0.83	1.10	0.50	0.92	0.84	1.01	0.09
Renal failure	1.50	1.27	1.78	0.00	0.90	0.82	1.00	0.05
Chronic Liver Disease	1.04	0.96	1.11	0.36	0.89	0.84	0.94	0.00
Diabetes	0.96	0.91	1.01	0.13	0.94	0.91	0.97	0.00
Hyperlipidemia	0.85	0.81	0.91	<0.001	0.96	0.93	0.99	0.01
Peripheral Vascular Disease	1.28	1.06	1.54	0.01	0.93	0.78	1.10	0.41
Outcomes								
Died	11.21	6.25	20.11	<0.001	4.88	2.36	10.11	0.00
Discharge location								
Routine discharge	0.27	0.22	0.32	<0.001	0.50	0.43	0.57	0.00
SNF discharge	6.46	5.42	7.69	<0.001	2.72	2.16	3.42	0.00
